# Laminar organization of spinal dorsal horn neurones activated by C- vs. A-heat nociceptors and their descending control from the periaqueductal grey in the rat

**DOI:** 10.1111/j.1460-9568.2007.05716.x

**Published:** 2007-08

**Authors:** Stella Koutsikou, Dilys M. Parry, Frankie M. MacMillan, Bridget M. Lumb

**Affiliations:** Department of Physiology, School of Medical Sciences, University Walk, University of Bristol BS8 1TD, UK

**Keywords:** Fos protein, midbrain, nociception, spinal cord

## Abstract

The periaqueductal grey can differentially control A- vs. C-nociceptor-evoked spinal reflexes and deep spinal dorsal horn neuronal responses. However, little is known about the control of A- vs. C-fibre inputs to lamina I and the lateral spinal nucleus, and how this correlates with the control of deeper laminae. To address this, the laminar distributions of neurones expressing Fos-like immunoreactivity were determined following preferential activation of A- or C-heat nociceptors, using fast or slow rates of skin heating, respectively, in the absence or presence of descending control evoked from the periaqueductal grey. In lamina I, numbers of Fos-positive neurones following both fast and slow rates of skin heating were reduced significantly following activation in the ventrolateral and dorsolateral/lateral periaqueductal grey. In contrast, in the deep dorsal horn (laminae III–VI), activation in both the ventrolateral and dorsolateral/lateral periaqueductal grey significantly reduced the numbers of Fos-positive neurones evoked by C- but not A-nociceptor stimulation. C- but not A-heat nociceptor activation evoked Fos bilaterally in the lateral spinal nucleus. Stimulation in the ventrolateral but not the dorsolateral/lateral periaqueductal grey significantly increased the numbers of Fos-positive neurones evoked by A- and C-nociceptor stimulation bilaterally in the lateral spinal nucleus. These data have demonstrated differences in the descending control of the superficial vs. the deep dorsal horn and lateral spinal nucleus with respect to the processing of A- and C-fibre-evoked events. The data are discussed in relation to the roles of A- and C-nociceptors in acute and chronic pain.

## Introduction

In physiological conditions, myelinated (A-fibre) and unmyelinated (C-fibre) peripheral nociceptors convey different qualities of the pain signal, i.e. first and second pain, respectively (Meyer *et al.*, 2006). Differences between A- and C-nociceptors have also been described in pathophysiological conditions, in particular in the development and maintenance of primary and secondary hyperalgesia (Baumann *et al.*, 1991; Fuchs *et al.*, 2000; Magerl *et al.*, 2001), which suggests a separation in their roles in chronic pain states.

The first relay in pain pathways activated by A- and C-nociceptors is the spinal dorsal horn and, as such, this represents an important site for the modulation of the pain signal. Descending control of spinal nociception, which originates from a number of supraspinal sites, is believed to be a major determinant of acute pain in different behavioural and emotional states (Bandler & Shipley, 1994; Lovick & Bandler, 2005). Importantly, it is now recognized that chronic pain states may also depend on descending controls that originate in the brain (Urban & Gebhart, 1999; Pertovaara, 2000; Porreca *et al.*, 2002; Ren & Dubner, 2002; Ossipov *et al.*, 2005; Suzuki & Dickenson, 2005) including, it is proposed, the periaqueductal grey (PAG) region of the midbrain (Pertovaara *et al.*, 1997; Monhemius *et al.*, 2001; Pertovaara & Wei, 2003; Vazquez *et al.*, 2007). Reports from this laboratory indicate that descending control from the PAG exerts differential control on A- vs. C-nociceptor-evoked withdrawal reflexes and deep dorsal horn neuronal responses (McMullan & Lumb, 2006a,b; Waters & Lumb, 2007). Potentially, this is of considerable significance, given differences in the roles of A- and C-nociceptors in acute and chronic pain.

The superficial dorsal horn has well-established roles in pain processing but little is known of the descending control of A- vs. C-fibre inputs to the superficial dorsal horn and how this correlates with the control of deeper laminae. Addressing this shortfall in our understanding was the primary aim of the current study.

Descending control of spinal nociception that can be evoked from either the ventrolateral (VL) or dorsolateral/lateral (DL/L) PAG is believed to operate as part of co-ordinated responses to emotional and environmental stressors, i.e. neurones in the DL/L-PAG co-ordinating active coping strategies in emergency situations and those in the VL-PAG co-ordinating passive coping strategies that operate during recuperation or after intense exercise (Bandler & Shipley, 1994; Lovick & Bandler, 2005). Our previous studies indicate that the DL/L and VL sectors of the PAG exert similar effects on pinch-evoked responses of dorsal horn neurones (Waters & Lumb, 1997). However, these experiments did not allow us to differentiate between activity evoked by A- vs. C-nociceptors and our observations were restricted to deep dorsal horn neurones. The second aim of the current study was to address both of these questions, i.e. (i) to determine the level of control exerted by the different functional columns of the PAG on A- vs. C-nociceptor-evoked activity and (ii) to compare effects exerted on superficial vs. deep dorsal horn neurones in the same animals.

## Materials and methods

### Animal preparation

Experiments were carried out on 94 adult male Wistar rats weighing 275–300 g. All experimental procedures were carried out in accordance with the UK Animals (Scientific Procedures) Act 1986.

Anaesthesia was induced using halothane (2.5% in O_2_) and the right jugular vein and carotid artery were cannulated to administer drugs and monitor arterial blood pressure, respectively. Following preparatory surgery, anaesthesia was monitored by assessing corneal reflexes and maintained by continuous intravenous infusion of sodium pentobarbital (∼31 mg/kg/h; Sigma, UK) or Rapinovet (∼30 mg/kg/h; Schering-Plough). Body temperature was maintained using a thermostatically controlled blanket. The head was fixed in a stereotaxic frame and a small hole was opened in the skull using a small drill fitted with a dental burr.

### Periaqueductal grey stimulation

Glass micropipettes (Harvard Apparatus Ltd, UK) were driven vertically into either the DL/L- or VL-PAG at approximately 7.6 mm caudal to bregma, 0.9 mm lateral to the midline and 4.4–4.5 mm deep to the cortical surface in order to reach the DL/L-PAG or 7.8 mm caudal to bregma and 5.25 mm deep to reach the VL-PAG.

Micropipettes were filled with 50 mm dl-homocysteic acid (DLH) (Sigma) solution containing pontamine sky blue dye to mark the injection sites. The concentration of DLH selected was the same as that used in previous studies of descending control from the PAG (McMullan & Lumb, 2006a,b) Three or six 30 nL pressure injections of DLH were given under microscopic guidance using a 1 µL glass syringe (SGE, Australia). Microinjections of DLH in the VL- or DL/L-PAG evoked decreases or increases, respectively, in the mean arterial blood pressure.

### Preferential activation of A- and C-heat nociceptors

A heating apparatus, which was a modified version of that described by Yeomans and colleagues (Yeomans & Proudfit, 1994, 1996; Yeomans *et al.*, 1996), was used to deliver slow or fast rates of skin heating to preferentially activate unmyelinated and myelinated heat nociceptors, respectively (McMullan *et al.*, 2004). In brief, heat from a sputter-coated projector bulb was focused onto a thin disk of blackened copper 4 mm in diameter, placed in firm, even contact with the dorsal (hairy skin) surface of the skin of the hindpaw. A custom-made t-type thermocouple (0.02 mm in diameter) was fixed in position between the surface of the copper disk and the skin and used to record skin temperature, which was digitized and captured at 50 samples/s on a PC running spike2 version 3.21 (Cambridge Electronic Design, Cambridge, UK). From a starting temperature of 30 °C, using a constant bulb voltage, fast rates of heating (7.5 ± 1 °C/s measured over 2 s from the start of heating) were used to preferentially activate myelinated heat nociceptors, whereas slow rates of heating (2.5 ± 1 °C/s measured over 4 s from the start of heating) were used to preferentially activate unmyelinated nociceptors. In order to prevent tissue damage, the three or six heat stimuli were applied to three different areas of the dorsal surface of the hindpaw with an 8 min gap between stimuli on different areas and a 24 min gap between stimuli on the same area. In addition, the skin heating was terminated by a feedback-controlled cut-off device. Cut-off temperatures to slow and fast rates of skin heating were 55 and 57 °C, respectively.

There is direct evidence from peripheral nerve recordings (Yeomans & Proudfit, 1996) that the same slow and fast rates of heating as used in the present study preferentially activate primary afferents that conduct in the C- and A-fibre range, respectively. Furthermore, evidence that slow and fast rates of skin heating preferentially activate C- and A-nociceptors, respectively, is provided by four lines of evidence from this laboratory: (i) differential capsaicin sensitivity of reflex responses evoked by slow, compared with fast, rates of skin heating (McMullan *et al.*, 2004), which is consistent with preferential activation of C-heat nociceptors, the majority of which express Transient Receptor Potential Channel V1 (TRPV1) receptors; (ii) differential dimethylsulphoxide sensitivity of responses evoked by fast, compared with slow, rates of skin heating (J.L. Leith, A.W. Wilson, L.F. Donaldson and B.M. Lumb, unpublished observation, and see Baumann *et al.*, 1991; Wilson *et al.*, 1999); (iii) response characteristics of dorsal horn neurones to slow vs. fast rates of skin heating closely resemble those of peripheral C- and A-nociceptive afferents, respectively (McMullan & Lumb, 2006b) and (iv) in contrast to dorsal horn neurones with C-fibre inputs, neurones lacking C-fibre inputs (C-negative neurones) do not respond to slow rates of skin heating (Leith *et al.*, unpublished observation).

### Experimental protocol

Each animal was assigned to one of the following experimental (A and B) or control (C and D) groups: (A) three or six applications of either fast (*n* = 15) or slow (*n* = 13) rates of skin heating in the absence of DLH microinjections; (B) three or six microinjections of DLH into either VL- (*n* = 20) or DL/L-PAG (*n* = 17) each followed by either fast (fast + VL-PAG, *n* = 9; fast + DL/L-PAG, *n* = 8) or slow (slow + VL-PAG, *n* = 11; slow + DL/L-PAG, *n* = 9) rates of skin heating; (C) six microinjections of DLH into either VL- (*n* = 6) or DL/L-PAG (*n* = 5) in the absence of skin heating and (D) sham animals that underwent preparatory surgery alone (*n* = 18). In each animal, microinjections and/or heat stimuli were delivered at 8 min intervals. Animals in group A did not have burr holes in the skull. However, there were no significant differences in Fos-positive neurones in groups C and D, suggesting that drilling the burr hole did not, in itself, alter spinal expression of Fos.

In initial experiments animals received six cycles of stimulation, which was later reduced to three. Comparisons in groups A and B revealed no significant differences (*P* > 0.05, Mann–Whitney) between the numbers of Fos-positive neurones in animals that received six or three cycles of stimulation. In group A, a response to slow rates of heating six and three heat ramps evoked 610 ± 92 and 633 ± 33 Fos-positive neurones, respectively, and 301 ± 27 and 362 ± 32, respectively, in response to fast ramps. As such, the data were pooled.

Following the preparatory surgery animals were allowed to stabilize for a minimum period of 2 h. During this period blood pressure and level of anaesthesia were monitored. Animals then underwent one of the protocols described above and were then maintained under anaesthesia for a further 2 h to allow expression of Fos protein in the dorsal horn of the spinal cord. The sham animals (group D) were maintained anaesthetized for 4 h following the preparatory surgery.

After this time, all animals were given an overdose of pentobarbital and perfused transcardially with mammalian Ringer (100 mL) followed by paraformadehyde (4% in 0.1 m phosphate buffer; 500 mL). The spinal cords (L3–L5) and brains were dissected out and post-fixed overnight in 4% paraformaldehyde. The tissue was then transferred to 30% sucrose for at least 24 h. Prior to sectioning, the spinal cord blocks were marked on the side contralateral to the peripheral stimulus using dye to aid orientation when viewed under the microscope.

### Tissue processing and immunocytochemistry

#### Periaqueductal grey injection sites

Sections (50 µm) of the PAG were cut on a freezing microtome for histological verification of pontamine sky blue dye injection sites.

#### Fos immunostaining

Transverse sections (40 µm) of the spinal cords were cut on a freezing microtome. Free-floating spinal cord sections were processed for Fos-like immunoreactivity (FLI) using a polyclonal rabbit Fos antibody (Santa Cruz Biotechnology; 1 : 5000 in 0.1 m phosphate buffer containing 1% bovine serum albumin, 1% normal goat serum and 0.1% triton X-100) for 48–72 h at 4 °C. Incubation in secondary biotinylated anti-rabbit antibody IgG (Sigma UK; 1 : 500 in 0.01 m phosphate-buffered saline with 0.1% triton X-100 (PBS-T)) was carried out for 1–2 h at room temperature (20°C). The sections were subsequently incubated in extravidin peroxidase (Sigma UK; 1 : 1000 in PBS-T) for 1–2 h and the peroxidase visualized using 3,3-diaminobenzidine (0.015%; Sigma UK) and glucose oxidase. Finally, all sections were mounted onto gelatine/chrome alum-coated microscope slides and cover-slipped using glycerol in carbonate buffer.

### Cell counting

Spinal cord sections were scanned at low magnification for Fos-positive nuclei in the dorsal horn and the 10 most heavily labelled sections were noted (Bullitt, 1990; Williams *et al.*, 1990). Fos-positive dorsal horn neurones were then counted at higher magnification in these sections and their locations assigned to the appropriate laminae of the spinal cord as distinguished under dark field illumination, i.e. lamina I, lamina II and laminae III–VI (deep dorsal horn). All of the sections from lumbar enlargements 3–5 were used to count Fos-positive nuclei in both the ispilateral and contralateral lateral spinal nucleus (LSN) and data are expressed as numbers of neurones per 200 sections.

### Data analysis

Data in bar charts are displayed as median ± interquartile range. Values in the text are means ± SEM. Comparisons between multiple groups were made using Kruskal–Wallis non-parametric variation of one-way anova. Data sets in which a significant change was observed were further compared using Dunn's multiple comparison post-hoc test. Comparisons between two groups were made using Mann–Whitney *U*-test. All statistical analysis was performed using GraphPad prism 4.0. *P* < 0.05 was considered to represent a significant difference.

## Results

### Expression of Fos in response to fast or slow rates of skin heating

**Fig. 1:**
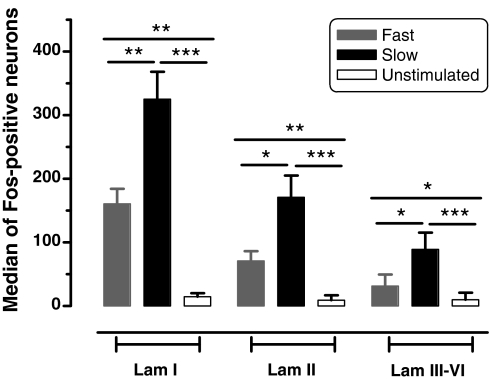
Fos-like immunoreactivity in the spinal dorsal horn in response to slow and fast rates of skin heating. Numbers of Fos-positive neurones in laminae (Lam) I and II (superficial dorsal horn), and Lam III–VI (deep dorsal horn) in response to fast (grey bars) and slow (black bars) rates of skin heating and in control animals (open bars). Values are median numbers per 10 40 µm sections and interquartile ranges. *P < 0.05, **P < 0.01, ***P < 0.001.

**Fig. 2:**
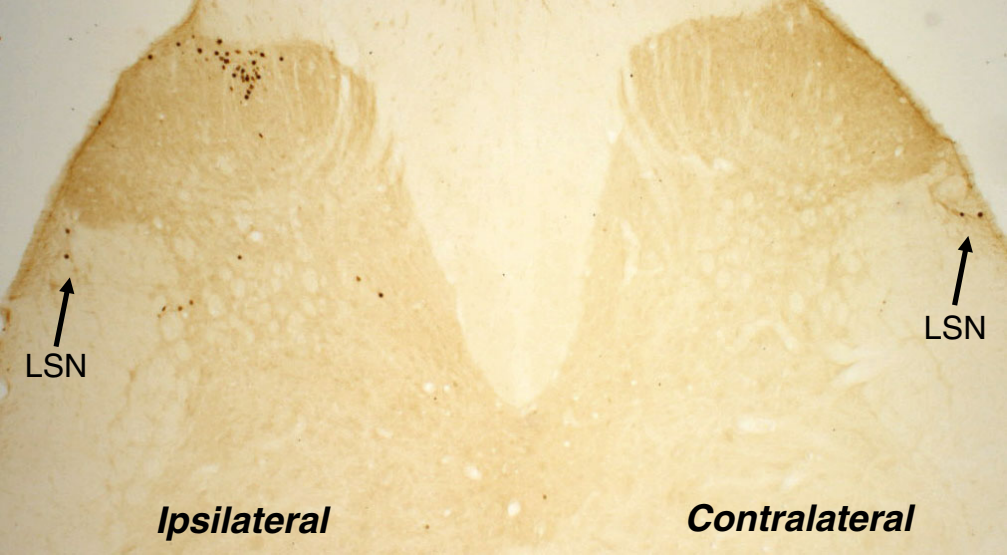
Fos-like immunoreactivity in the spinal cord in response to slow rates of skin heating. Photomicrograph of dorsal half of spinal cord to show Fos-positive neurones in the ipsilateral dorsal horn and bilaterally in the lateral spinal nucleus (LSN).

**Fig. 6:**
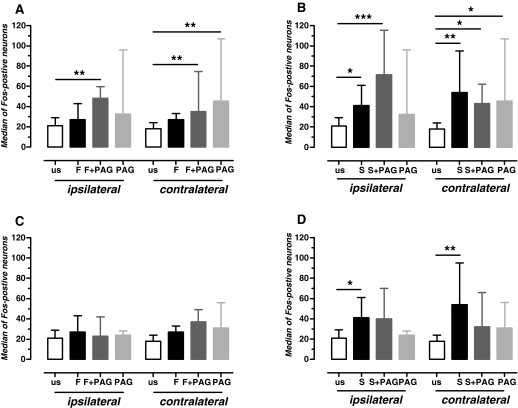
Fos-like immunoreactivity in the lateral spinal nucleus in response to different rates of skin heating and the effects of activation in the periaqueductal grey (PAG). Histograms of median numbers per 10 40 µm sections of Fos-positive neurones in response to fast (A and C) and slow (B and D) rates of skin heating with and without PAG (A and B, ventrolateral; C and D, dorsolateral/lateral) activation. Statistical comparisons (Kruskal–Wallis) were made between all groups in each of A–D. Lack of asterisks indicates no significant difference. us, unstimulated controls; F, fast rate of skin heating; F + PAG, fast rate of skin heating plus activation in PAG; PAG, activation in the PAG alone; S, slow rate of skin heating; S + PAG, slow rate of skin heating plus activation in PAG. *P < 0.05, **P < 0.01, ***P < 0.001.

In the ipsilateral but not the contralateral lumbar segments 3–5, both slow and fast rates of skin heating of the hindpaw dorsum evoked significantly more Fos-positive neurones in laminae I, II and III–VI (*P* at least < 0.05) than in unstimulated controls (Fig. 1). In both the superficial (laminae I and II) and deep (laminae III–VI) dorsal horn there were significantly more Fos-positive neurones in response to slow compared with fast rates of skin heating (*P* < 0.01, *P* < 0.05 and *P* < 0.05, respectively; Fig. 1). Consistent with previous studies that allowed 2 h for the induction of Fos protein (Coggeshall, 2005), there were significantly more neurones in the superficial compared with the deep dorsal horn in response to noxious heat stimulation (*P* < 0.001; Mann–Whitney test, comparison not shown). Compared with unstimulated animals (ipsilateral, 20.7 ± 4.1; contralateral, 17.0 ± 3.3; Fig. 6), slow but not fast rates of skin heating evoked significantly more Fos-positive neurones bilaterally in the LSN (ipsilateral, 46.7 ± 8.4, *P* < 0.05; contralateral, 60.9 ± 10.0, *P* < 0.001; see Figs 2 and 6). There was no significant difference in the numbers of Fos-positive LSN neurones ipsilateral and contralateral to the noxious stimulus.

### Effects of dl-homocysteic acid microinjections in the periaqueductal grey on Fos expression in the dorsal horn in response to fast and slow rates of skin heating

**Fig. 3:**
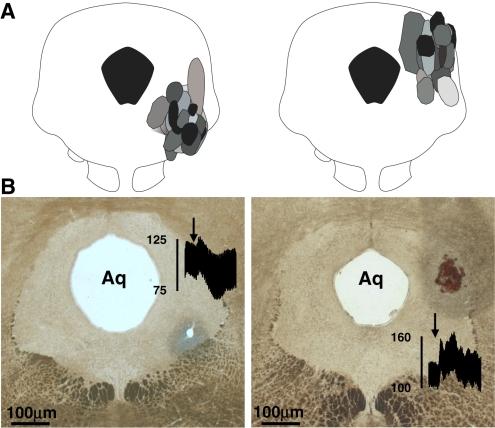
Locations of dl-homocysteic acid injection sites in ventrolateral (VL) and dorsolateral/lateral (DL/L) periaqueductal grey (PAG). (A) Representative transverse sections to illustrate all injection sites in the VL- and DL/L-PAG. Locations of injections in control (group C) and experimental (group B) animals are shown in black and shades of grey, respectively. (B) Photomicrographs of individual examples of injection sites in VL- and DL/L-PAG. Insets are examples of evoked changes in blood pressure (mmHg). Aq, Aqueduct.

**Fig. 4:**
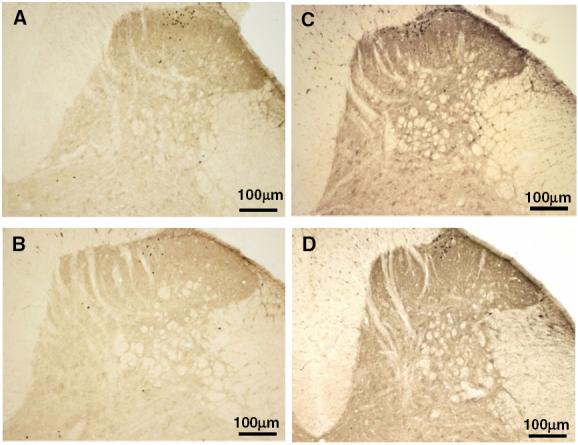
Effects of activation in the periaqueductal grey (PAG) on spinal Fos-like immunoreactivity. Photomicrographs of Fos-like immunoreactivity in response to fast (A and B) and slow (C and D) rates of skin heating in the absence (A and C) and presence (B and D) of evoked descending control from the ventrolateral PAG.

**Fig. 5:**
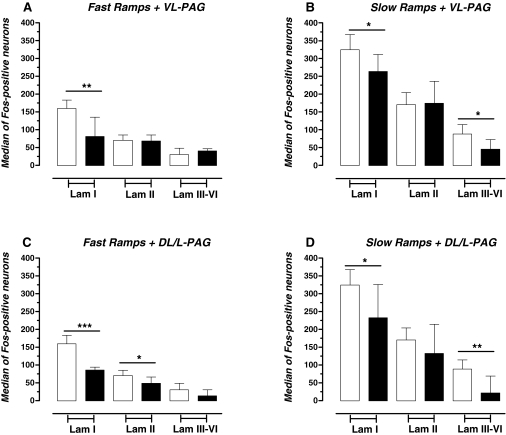
Effects of activation in the periaqueductal grey (PAG) on Fos-like immunoreactivity in the dorsal horn. Histograms of median numbers per 10 40 µm sections of Fos-positive neurones in laminae (Lam) I and II and Lam III–VI in response to fast (A and C) and slow (B and D) rates of skin heating with and without the influence of descending control exerted by different sectors of the PAG [A and B, ventrolateral (VL) PAG; C and D, dorsolateral/lateral (DL/L) PAG]. Numbers of Fos-positive neurones in the absence and presence of descending control from the PAG are shown by open and filled bars, respectively. *P < 0.05, **P < 0.01, ***P < 0.001.

Locations of depressor sites in the VL-PAG (*n* = 25) and pressor sites in the DL/L-PAG (*n* = 22) in experimental (group B) and control (group C) animals are illustrated in Fig. 3, in which the symbols represent the spread of pontamine sky blue dye in the DL/L- or VL-PAG. Individual examples of the effects of DLH injection in the PAG on FLI in the dorsal horn are illustrated in Fig. 4 and the laminar distributions of Fos-positive neurones in the presence and absence of evoked descending control in Fig. 5.

#### Effects on Fos-like immunoreactivity in the superficial dorsal horn (laminae I and II)

In lamina I, FLI evoked by both slow and fast rates of skin heating was reduced significantly following activation in the VL- and DL/L-PAG (Fig. 5): (i) slow ramps from 322 ± 15.2 to 262.5 ± 16.6 (*P* < 0.05, VL-PAG) and from 322 ± 15.2 to 247.4 ± 28.2 (*P* < 0.05, DL/L-PAG), and (ii) fast ramps from 158.7 ± 8.8 to 102.4 ± 13.3 (*P* < 0.01, VL-PAG) and from 158.7 ± 8.8 to 82.6 ± 6.7 (*P* < 0.001, DL/L-PAG).

In lamina II, numbers of Fos-positive neurones in response to fast, but not slow, rates of skin heating were decreased significantly (from 78.9 ± 9.1 to 53.1 ± 6.9, *P* < 0.05) following activation in DL/L-PAG (Fig. 5C and D). In contrast, numbers of Fos-positive neurones in response to slow rates of skin heating were unaltered by activation in either the VL- or DL/L-PAG (Fig. 5B and D).

#### Effects on Fos-like immunoreactivity in the deep dorsal horn (laminae III–VI)

In the deep dorsal horn, FLI in response to slow rates of skin heating was reduced significantly following injection of DLH at sites in both the VL- and DL/L-PAG (Fig. 5B and D) [from 88.5 ± 10.5 to 48.0 ± 8.5 (*P* < 0.05) and from 88.5 ± 10.5 to 40.9 ± 11.4 (*P* < 0.01), respectively]. FLI in response to fast rates of skin heating was unaltered following DLH injection at sites in either the VL- or DL/L-PAG (Fig. 5A and C).

### Effects of dl-homocysteic acid microinjections in the periaqueductal grey on Fos expression in lateral spinal nucleus in response to slow and fast rates of skin heating

As described above and illustrated in Fig. 6A, in the absence of PAG stimulation fast rates of skin heating failed to evoke significantly more Fos-positive neurones when compared with unstimulated controls. However, activation in the VL-PAG (at sites shown in Fig. 3) resulted in significant increases (*P* < 0.01) in the numbers of Fos-positive neurones evoked by fast rates of skin heating bilaterally in the LSN (Fig. 6A). Effects of activation in the VL-PAG on FLI in response to slow rates of skin heating are shown in Fig. 6B. VL-PAG stimulation had no significant effects on FLI evoked by the heat stimulus but it did increase FLI in the contralateral LSN compared with unstimulated control levels. Activation in the DL/L-PAG (at sites shown in Fig. 3) had no effect on the numbers of Fos-positive neurones in either the ipsilateral or contralateral LSN (Fig. 6C and D).

### Control experiments

Numbers of Fos-positive neurones in the dorsal horn in control animals (group C) in which DLH was injected into pressor sites in the DL/L-PAG (*n* = 5) or depressor sites in the VL-PAG (*n* = 6) at sites shown in Fig. 3 were not significantly different (*P* > 0.05) from non-injected sham animals (group D, data not illustrated). Injection of DLH into the VL- but not the DL/L-PAG significantly increased (from 17.0 ± 3.3 to 60.2 ± 21.3; *P* < 0.05) the numbers of Fos-positive neurones in the contralateral but not the ipsilateral LSN (Fig. 6).

## Discussion

By combining the expression of Fos protein, to identify populations of spinal neurones excited by preferential activation of A- or C-heat nociceptors, with neuronal stimulation at physiologically identified sites in the midbrain we have been able to determine the level of control exerted by functionally distinct columns of the PAG on A- vs. C-fibre-evoked activity in different regions of the spinal cord in individual animals. The novel findings, as determined by comparisons of numbers of activated neurones, suggest that (i) descending control from the PAG differentially inhibits C- vs. A-fibre-evoked events in the deep dorsal horn but inhibits both C- and A-fibre-evoked events in lamina I; (ii) there are no differences in the nature of control exerted by the DL/L- vs. VL-PAG on evoked activity in lamina I or the deep dorsal horn, although differences were observed in lamina II, and (iii) in the absence of descending control the LSN is activated bilaterally by C- but not A-nociceptive inputs and that activation of the VL-PAG, but not the DL/L-PAG, significantly increases A-nociceptor-evoked FLI bilaterally in the LSN.

### Organization of A- and C-heat nociceptive input into the dorsal horn and its descending control

Fast and slow rates of skin heating evoked FLI throughout the dorsal horn with no difference in the overall pattern of labelling in superficial and deep laminae. However, in all laminae there were approximately twice as many neurones activated by C- compared with A-fibre stimulation, which may reflect a greater spinal divergence of C-fibre input (Fitzgerald & Wall, 1980; Tattersall *et al.*, 1986; Cervero & Tattersall, 1987), including that resulting from polysynaptic inputs and/or differences in the peripheral innervation density of A- and C-fibre nociceptors in the hindpaw dorsum. Alternatively, the longer slow ramp stimulus, which would impart greater heat energy, may account for the increased numbers of Fos-positive neurones compared with those activated by fast heat ramps.

#### Control of A- and C-fibre input to the superficial dorsal horn from dorsolateral/lateral and ventrolateral periaqueductal grey

Descending control of nociceptive responses of superficial dorsal horn neurones from brain stem sites, including the PAG, has been described in electrophysiological and Fos-expression studies (Willis, 1988; Millan, 2002; Coggeshall, 2005). However, few studies have compared the effects on A- vs. C-nociceptor-evoked responses. Here we show that the PAG exerts non-selective inhibitory control of A- and C-nociceptor-evoked activity in lamina I, with no difference in the level of control exerted by the VL- and DL/L-PAG, which is consistent with previous reports (Mokha *et al.*, 1987) of similarities in the effects of lesions in VL- and DL/L-PAG on hypothalamic control of trigeminal lamina I neurones.

An unexpected finding was the apparent lack of effect of descending control from the PAG on the number of nociceptor-evoked Fos-positive neurones in lamina II. Few Fos-expression studies have discriminated between descending control of laminae I and II (Jones & Light, 1990; Gogas *et al.*, 1991; Wei *et al.*, 1999; Coggeshall, 2005), which makes direct comparisons difficult. However, the available evidence would predict a reduction in activated neurones. Consistent with a spinal modulatory role, there are few projection neurones in lamina II, rather a heterogeneous population of inhibitory and excitatory interneurones with different chemical phenotypes (Millan, 1999; Morris *et al.*, 2004). It is possible therefore that the apparent lack of effect arises from mixed excitation and inhibition of different populations of interneurones, resulting in any net effect on overall numbers being obscured. The observation that DLH injection into the PAG, in the absence of peripheral stimulation, did not evoke FLI in lamina II suggests that post-synaptic excitation of a sub-population of neurones is unlikely to contribute to any increase in numbers of labelled neurones; it does not, however, preclude sub-threshold post-synaptic changes or the possibility of pre-synaptic facilitation of input arriving in nociceptive afferents.

#### Control of A- and C-fibre input to the deep dorsal horn from dorsolateral/lateral and ventrolateral periaqueductal grey

Numerous studies describe descending control from the PAG of nociceptive responses of deep dorsal horn neurones (Willis, 1988; Millan, 2002). Importantly, the finding that the PAG has differential effects on C- vs. A-nociceptor-evoked spinal events is consistent with previous electrophysiological studies in which inhibitory control from the PAG was found to target C- as opposed to A-nociceptor-evoked spinal reflexes (McMullan & Lumb, 2006a) and dorsal horn neurones driven by C-nociceptive inputs (Waters & Lumb, 1997). The observation that equivalent effects can be evoked from both the DL/L- and VL-PAG is also consistent with our previous findings (Waters & Lumb, 1997). The current study used expression of Fos protein to determine the influences of descending control on the regional distribution of whole populations of neurones activated by C- or A-nociceptor stimulation. As such, the data do not shed light on spinal mechanisms underlying differential control. However, the decrease in total numbers of neurones activated by slow heat ramp stimulation does support the view that it may be mediated by post-synaptic targeting of a sub-population of neurones with C-fibre inputs (McMullan & Lumb, 2006b). The more robust responses of deep dorsal horn neurones to fast compared with slow rates of skin heating (McMullan & Lumb, 2006b) might well account, at least in part, for their resistance to descending control observed in the present study.

### Organization of A- and C-heat nociceptive input into the lateral spinal nucleus and its descending control

In agreement with other studies that used noxious heat stimuli (Olave & Maxwell, 2004), neurones were found bilaterally in the LSN, with no significant difference in ipsilateral and contralateral FLI. However, the observation that slow, but not fast, rates of skin heating evoked significant numbers of Fos-positive neurones in the LSN suggests that this region is targeted by C-, as opposed to A-, nociceptor inputs. This may account for the findings of earlier electrophysiological studies (Menetrey *et al.*, 1980) in which LSN neurones were driven by deep noxious stimuli, which are likely to be mediated by C-nociceptors, and had poorly defined cutaneous receptive fields. Interestingly, Menetrey *et al.* (1980) reported that a large proportion (40%) of LSN neurones project bilaterally to supraspinal structures, including the PAG, and they suggested that the LSN might represent a relay in unmyelinated pathways between the periphery and brain. Our own studies (Lumb *et al.*, 2004) also report that projections of a large proportion (approx. 40%) of C-nociceptor-activated LSN neurones target the VL-PAG. Taken together, these findings could account for the bilateral activation of neurones in the VL-PAG following unilateral C-fibre stimulation in the periphery (Lumb *et al.*, 2002; Lumb, 2002).

An unexpected finding was that activation in the VL- but not the DL/L-PAG significantly increased the numbers of A-nociceptor-activated neurones bilaterally in the LSN. Given that, in the absence of PAG activation, fast ramp stimulation did not evoke significant FLI in the LSN bilaterally and that VL-PAG activation alone did not always evoke significant FLI in the LSN, this observation suggests that the VL-PAG can facilitate A-fibre nociceptive processing in the LSN, many of whose neurones project to supraspinal sites (Kenshalo *et al.*, 1980; Parry *et al.*, 2002; Olave & Maxwell, 2004). The functional significance of this finding is unclear but it is interesting to speculate that the PAG may up-regulate nociceptive input to brainstem control centres via an action on relays in the LSN.

### Functional significance

There is much debate about the relative contributions of superficial and deep dorsal horn neurones to sensori-discriminative vs. affective aspects of pain signalling. There is little doubt, however, that neurones in the deep dorsal horn accurately encode the intensity of noxious stimuli (McMullan & Lumb, 2006b). As such, preferential suppression of C-nociceptor-evoked responses in the deep dorsal horn, as reported here and in our previous electrophysiological studies (McMullan & Lumb, 2006a; Waters & Lumb, 2007), has important implications as it is the slowly conducted, poorly tolerated and therefore arguably distracting component of the nociceptive message that would be depressed at the spinal level and the high-resolution, rapidly conducted component that would be left intact. Our data indicate that this combination of effects could operate as part of both active and passive coping strategies that are co-ordinated by the DL/L- and VL-PAG, respectively. Therefore, in both situations differential control of A- vs. C-fibre-evoked events in the deep dorsal horn could preserve the detailed information of changes in the external environment that can drive motivational behaviours and accurately direct motor activity, whilst depressing those components of the nociceptive message that are less useful in terms of survival and recuperation.

It is now evident that superficial and deep dorsal horn neurones have different roles in spino-bulbo-spinal mechanisms of central sensitization and hyperalgesia. An emerging view (Fields, 2004; Mantyh & Hunt, 2004; Vanegas & Schaible, 2004) is that a sub-population of supraspinally projecting lamina I neurones sets the level of descending control of deep dorsal horn neurones. It is proposed that dynamic changes in this control, driven by nociceptive inputs from damaged or inflamed peripheral tissue, may contribute to the transition from acute to chronic pain (Khasabov *et al.*, 2002; Suzuki *et al.*, 2002). The current findings indicate that differences in nociceptive processing in the superficial and deep dorsal horns extend to the descending control of their A- and C-nociceptor input, and that non-selective control of the superficial dorsal horn has the capacity to trigger differential control in the deep dorsal horn. Future studies of descending control of A- vs. C-fibre-evoked spinal nociception that characterize superficial dorsal horn neurones as excitatory or inhibitory interneurones, or as projection neurones, will significantly advance our understanding of the role of spino-bulbo-spinal loops in setting the gain of acute and chronic spinal nociception.

## Abbreviations

DLH: dl-homocysteic acid
DL/L: dorsolateral/lateral
FLI: Fos-like immunoreactivity
LSN: lateral spinal nucleus
PAG: periaqueductal grey
VL: ventrolateral.
